# Isolation and Characterization of Cellulose Nanocrystals from Oil Palm Mesocarp Fiber

**DOI:** 10.3390/polym9080355

**Published:** 2017-08-11

**Authors:** Buong Woei Chieng, Syn Huey Lee, Nor Azowa Ibrahim, Yoon Yee Then, Yuet Ying Loo

**Affiliations:** 1 Department of Chemistry, Faculty of Science, Universiti Putra Malaysia, UPM Serdang, Selangor 43400, Malaysia; music_huey@yahoo.com (S.H.L.); norazowa@upm.edu.my (N.A.I.); 2 Materials Processing and Technology Laboratory, Institute of Advanced Technology, Universiti Putra Malaysia, UPM Serdang, Selangor 43400, Malaysia; 3 Department of Pharmaceutical Chemistry, School of Pharmacy, International Medical University, No. 126, Jalan Jalil Perkasa 19, Bukit Jalil, Kuala Lumpur 57000, Malaysia; thenyoonyee@imu.edu.my; 4 Department of Food Science, Faculty of Food Science and Technology, Universiti Putra Malaysia, UPM Serdang, Selangor 43400, Malaysia; yuetying88@gmail.com

**Keywords:** mesocarp fiber, oil palm, cellulose

## Abstract

The aim was to explore the utilization of oil palm mesocarp fiber (OPMF) as a source for the production of cellulose nanocrystals (CNC). OPMF was first treated with alkali and then bleached before the production of CNC by acid hydrolysis (H_2_SO_4_). The produced materials were characterized using Fourier transform infrared (FTIR) spectroscopy, X-ray diffraction (XRD), thermogravimetric analysis (TGA), a scanning electron microscope (SEM) and a transmission electron microscope (TEM). It was proven that acid hydrolysis can increase the crystallinity of bleached OPMF and reduce the dimension of cellulose to nano scale. Changes in the peaks of the FTIR spectrum at 2852 (C-H stretching), 1732 (C=O stretching) and 1234 cm^−1^ (C-O stretching) indicated that the alkali treatment completely removed hemicelluloses and lignin from the fiber surface. This can be seen from the thermogram obtained from the TGA characterization. Morphological characterization clearly showed the formation of rod-shaped CNCs. The promising results prove that OPMF is a valuable source for the production of CNC.

## 1. Introduction

Palm oil trees (*Elaeis guineesis*) originated from West Africa, are one of the major agricultural crops contributing to Malaysia’s economic growth. Throughout the years, Malaysia has harnessed the benefits of the oil palm industry and has become important in the industry internationally [1]. The biomass generated from Malaysia’s oil palm industry was 83 million tonnes in 2012, and is expected to grow to over 100 million tonnes by 2020 [2]. This biomass includes the oil palm empty fruit bunches (OPEFB), palm kernel shells (PKS), oil palm mesocarp fibers (OPMF), palm oil mill effluent (POME), oil palm trunks (OPT), oil palm leaves (OPL) and oil palm fronds (OPF).

The oil palm biomass is mainly composed of lignin, cellulose (α-cellulose) and hemicellulose, and the composition varies accordingly. Lignin is an amorphous heteropolymer with a complex structure. It provides a barrier to the cell wall as it is rigid, insoluble in water, impermeable, and resistant to microbial attack and oxidative stress [3]. Cellulose is an insoluble polysaccharide with a degree of polymerization of about 10,000, consisting of linear chains of glucopyranose units linked by a β-1,4 glycosidic bond. It has a general formula of (C_6_H_10_O_5_)_n_. Hemicellulose is a complex mixture of several polysaccharides such as pentoses (xylose, arabinose), hexoses (mannose, glucose, galactose) and acylated sugars. It has an average molecular weight of approximately 30,000 g/mol, and is a component of the cell wall [4].

Oil palm mesocarp fiber (OPMF) is the biomass residue obtained from the oil palm fruits after the extraction of palm oil. It is usually left as waste in the palm oil mill or utilized in generating power as boiler fuel producing steam. However, such utilization has created a pollution problem to the environment. Generally, OPMF consists of fruit fiber, crushed kernels and shells [5]. As reported by Saka et al. [6], OPMF is made up of 39.5 wt % of cellulose, 32.8 wt % of lignin, 9.8 wt % of hemicelluloses, 9.3 wt % of ash and 8.6 wt % of extractives. Being rich in lignocellulose, there is potential for the production of nanocellulose from OPMF for various applications, particularly for nanocomposite materials as reinforcing filler for the automotive industry, predominantly for interior applications, construction, electronics, cosmetics, packaging and also for biomedicine purposes [7].

Nanocellulose is a general term for the cellulosic particles with nano-scale structural dimensions. Recently, the preparation of nanocellulose has garnered interest for being biodegradable and nontoxic, and the extraordinary properties brought by the nano-size effect [8]. Nanocellulose can be further divided into three types of materials, namely cellulose nanocrystals (CNCs), cellulose nanofibrils (CNFs), and bacterial cellulose (BC). Each of the three types of nanocellulose requires different methods of extraction from the cellulose sources and possesses distinct properties, which dictate their applicability and functionality [9]. The production of CNCs from the source fibers consists of three main parts: pretreatment of the fibers, isolation of CNCs, and post-treatment of hydrolyzed celluloses. Pretreatment of fibers includes the alkali treatment and bleaching treatment. Isolation of CNCs usually involves acid hydrolysis. Other methods include enzymatic hydrolysis, TEMPO oxidation and the use of ionic liquid for the isolation of CNCs. The post-treatment of hydrolyzed celluloses includes purification and sonication [10].

The extraction of CNCs can be done by using various fibers ranging from fresh fibers to biomass. In the oil palm industry, research has focused on the production of CNCs from OPEFB [11,12,13,14]. Recently, research on the CNC isolated from oil palm trunk (OPT) using acid hydrolysis method has been reported by Lamaming et al. [7] and Mazlita et al. [15]. However, no research has been done on CNC isolated from other parts of oil palm biomass, especially OPMF. For this reason, the purpose of this research was to isolate and characterize the CNCs extracted from OPMF. The properties of the CNC isolated from OPMF were characterized using Fourier transform infrared (FTIR) spectroscopy, Thermogravimetric analysis (TGA), X-ray diffraction (XRD) analysis, a Scanning electron microscope (SEM) and a Transmission electron microscope (TEM).

## 2. Materials and Methods

### 2.1. Materials

Oil palm mesocarp fiber (OPMF) was obtained from FELDA Serting Hilir Palm Oil Mill (Kuala Lumpur, Malaysia). Before use, OPMF was washed by soaking in distilled water for 24 h, then rinsed with hot water (60 °C) and acetone prior to drying at 60 °C in an oven. This process was carried out to remove dirt adhering to the fiber surface. The dried fiber was then ground, and sieved into a particle size of <150 μm. Sodium chlorite was purchased from Acros Organics (Kuala Lumpur, Malaysia). Acetic acid glacial 99.7% was purchased from SYSTERM (Selangor, Malaysia). Sodium hydroxide, potassium hydroxide 85%, and sulphuric acid 98% were purchased from R&M Chemicals (Selangor, Malaysia). All chemicals were used as received without further purification.

### 2.2. Preparation of Cellulose Fibers

OPMF smaller than 150 µm was treated in reflux condition with 4 wt % NaOH solution at 80 °C for 3 h. This alkali treatment was conducted three times, and after each treatment, the fibers were filtered and washed with distilled water. The alkali extraction is performed to remove alkali-soluble components, in essence, lignin and hemicelluloses that dissolve in the solution.

The alkali-treated fibers were then bleached three times at 80 °C for 4 h by using equal parts of acetate buffer (solution of 2.7 g NaOH and 7.5 mL of glacial acetic acid in 100 mL of distilled water), aqueous chlorite (1.7% *w*/*v*), and distilled water. The bleached fibers were subsequently filtered, washed with distilled water, and air-dried [16]. The purpose of this bleaching treatment is to break down phenolic compounds or molecules with chromophoric groups present in lignin, and to remove the by-products, and thus whitening the material.

### 2.3. Extraction of Cellulose Nanocrystals

Acid hydrolysis was conducted on the obtained cellulose fibers using 65 wt % H_2_SO_4_ at 45 °C for 45 min. The hydrolysis treatment was to remove the amorphous regions of the cellulose and release cellulose nanocrystals from the cellulose substrate. The hydrolyzed cellulose sample was later diluted with cold distilled water to interrupt the reaction. It was then centrifuged three times at 10,000 rpm and 10 °C for 10 min, to remove excess acid and water-soluble fragments. Dialysis against distilled water until a constant pH was then performed to remove any free acid molecules. Sonication was then carried out to disperse nanocrystals. The resulting suspension was dried in a freeze drier and kept for further use.

### 2.4. Characterizations

#### 2.4.1. Chemical Composition of Oil Palm Mesocarp Fiber

The chemical compositions of the OPMF were determined according to the standard procedures described in our previous paper [17]. This allowed determining the weight fraction of cellulose, hemicellulose and lignin contents in OPMF. The average values of three replicated samples were recorded.

#### 2.4.2. Fourier Transform Infrared (FTIR) Spectroscopy

The FTIR spectra were recorded on an attenuated total reflection Fourier transform infrared (ATR-FTIR) by Shimadzu IRTracer-100 FTIR Spectrophotometer (Kyoto, Japan) to examine the changes in functional groups induced by various treatments. The FTIR spectral analysis was performed within a range of 400–4000 cm^−1^.

#### 2.4.3. X-ray Diffraction (XRD) Analysis

The XRD patterns were obtained to determine the crystallinity index of the CNCs obtained. The samples were scanned at 2°/min with a 2θ angle range from 2° to 60° by using an X-ray diffractometer (Philips/X’Pert Pro Panalytical-PW 3040/60 MPD, Almelo, The Netherlands). The crystallinity index value was computed to quantify the crystallinity of the samples. The crystallinity index (C_Ir_) is defined by:

(1) $$C_{\mathrm{Ir}}\;\left(\%\right)=\;\frac{\left(I_{002}-I_{\mathrm{am}}\right)}{I_{002}}\times 100$$

where *I*_002_ is the peak intensity corresponding to crystalline region and *I*_am_ is the peak intensity of the amorphous region.

#### 2.4.4. Thermogravimetric Analysis (TGA)

TGA measures the changes in weight of a sample as a function of temperature and/or time. The thermo-stability property of the sample prepared was analyzed by using Perkin Elmer Thermal Analyzer (Waltham, MA, USA). About 9 mg of sample was heated at a rate of 10 °C/min with nitrogen gas as carrier at flow rate of 50 mL/min. The weight percentage of residue was recorded to determine the weight losses of the sample after heating.

#### 2.4.5. Scanning Electron Microscopy (SEM)

SEM was used to study the effects of various treatments on the morphology of the samples. The SEM micrographs of OPMF, alkaline-treated OPMF, bleached OPMF and CNC produced from OPMF were recorded by using a JEOL (Tokyo, Japan) JSM-6400 scanning electron microscope operating at 15 kV accelerating voltage. The samples were prepared by placing them on a metal holder and coating them with gold by a Bio-rad (Hercules, CA, USA) coating system for 3 min to ensure good conductivity prior to analysis.

#### 2.4.6. Transmission Electron Microscopy (TEM)

TEM was used to study the structure and size of CNCs produced from OPMF. CNCs were dropped in a 300-mesh copper carbon grid, draining the excess of water during sample preparation. The CNCs were contrasted with a 2% (*w*/*v*) uranyl acetate solution and analyzed by a JEOL (Tokyo, Japan) JEM-1011 transmission electron microscope.

## 3. Results

### 3.1. Chemical Composition of Oil Palm Mesocarp Fiber

The chemical composition of OPMF and treated OPMF was determined and the results are summarized in Table 1. The OPMF consisted of 32.22% of cellulose, 31.62% hemicellulose and 23.89% lignin. This showed that OPMF could be a good source of cellulose. OPMF is particularly of interest due to its recognized potential as a source of biomass such as cellulose, resulting in an environmentally friendly and cost effective alternative source. The effect of alkaline and bleaching treatments on OPMF composition was that the cellulose content increased as expected. The treatments were efficient at removing most of the hemicellulose and lignin, resulting in high cellulose content. The treated OPMF contained 81.11% cellulose, 12.65% hemicellulose, and 5.09% lignin.

**Figure 1:**
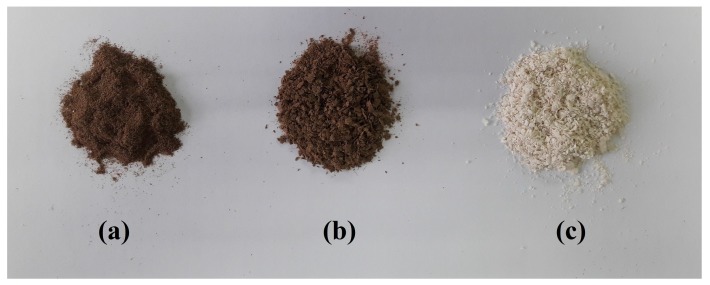
Photographs of (**a**) OPMF, (**b**) alkaline treated OPMF and (**c**) bleached OPMF.

Figure 1 shows the physical appearances of OPMF, alkaline treated OPMF and bleached OPMF. The color of the OPMF changed as a result of the treatments. As shown in Figure 1a, OPMF had a brown color. The alkali treatment did not alter much the color of OPMF, as alkaline treated OPMF showed very similar color to that of OPMF. In contrast, the bleaching treatment produced fiber that was relatively white in color (Figure 1c). The change in color of fiber after treatments is related to the degradations of cellulose, hemicellulose, and lignin substances [18]. The degradation process is normally accompanied by weight loss and alteration of chemical compositions of fiber.

### 3.2. Fourier Transform Infrared (FTIR) Analysis

**Figure 2:**
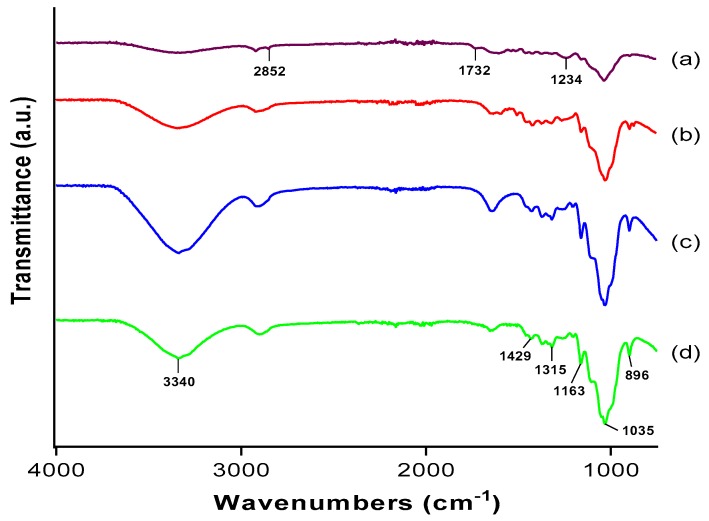
Fourier transform infrared (FTIR) spectra of (a) OPMF, (b) alkaline treated OPMF, (c) bleached OPMF and, (d) cellulose nanocrystals (CNC).

The FT-IR technique was carried out to study the functional groups present in OPMF and to examine the changes that occurred due to various treatments. Being lignocellulosic, OPMF is composed of mainly hemicellulose, lignin and celluloses, meaning that the main composition of the fiber includes alkanes, esters, aromatics, ketones, and alcohols with different oxygen-containing functional groups [19]. Figure 2 shows the spectra of (a) OPMF, (b) alkaline treated OPMF, (c) bleached OPMF and, (d) acid hydrolysed OPMF (CNC). The peaks at 3340 cm^−1^ in all of the spectra correspond to O–H stretching and reflect the tendency of OPMF to be hydrophilic [7]. The intensity of the peak inclines when treated with alkaline, due to the increase of OH concentration as the alkaline reduced the hydrogen bonding in cellulosic hydroxyl groups [20]. This reaction can also explain the diminishing of the peak at 2852 cm^−1^, present only in the spectrum of the OPMF which correspond to C–H stretching. 

Another peak that is present only in the spectrum of OPMF is at 1732 cm^−1^, corresponding to C=O stretching of carbonyl functional groups from hemicellulose and lignin fractions [21]. The C=O stretching disappears after alkaline treatment due to removal of carboxylic groups, which may be traces of fatty acids present on the fiber surface. The peak at 1234 cm^−1^ in the spectrum of OPMF, corresponding to syringyl ring and C–O stretching of lignin and xylan, shows the decline in its intensity and then diminishes, confirming the removal of lignin and a small part of hemicellulose from the OPMF after various treatments [15].

The peak at 1163 cm^−1^ in the CNC spectrum revealed the presence of a sulphated group (SO_2_) probably due to sulfonation of cellulose occurring during the acid hydrolysis process using sulphuric acid.

In the CNC spectrum, the peaks are present at 1429, 1315, 1035, and 896 cm^−1^, representing typical cellulose absorption peaks. Each peak can be assigned respectively as CH_2_ bending, CH_2_ rocking, C–O stretching and C–H or CH_2_ bending. Additionally, the peak at 896 cm^−1^ can be attributed to the typical structure of cellulose due to the β-glycosidic linkages of the glucose ring of the cellulose chain [7]. The presence of glycosidic linkages is evidence of a cellulose structure as these linkages bond anomeric carbon atom of saccharides to form polysaccharides. Complementing these SEM results, the alkaline treatment and bleaching process of OPMF successfully showed the presence of a pure cellulose phase (lignin and hemicellulose being removed) and the cleavage of a glycosidic bond to break long chain of cellulose into nanocrystallite.

Overall, the typical absorption band present in the extracted cellulose and CNC from OPMF spectra is similar to that obtained from coconut fiber at 1431, 1319, 1034, and 897 cm^−1^ [22].

### 3.3. X-ray Diffraction (XRD) Analysis

The XRD patterns were obtained to determine the crystalline index of the OPMF, alkaline treated OPMF, bleached OPMF and CNC obtained from the OPMF. Crystallinity index (CrI) refers to the ratio of the crystalline to the amorphous regions of cellulose. Cellulose crystallinity has been used to determine the elasticity, thermal stability, absorptive capacity and other physical properties of the fiber which are important for industrial purposes. An increase in crystallinity is expected to increase the stiffness and rigidity, and therefore strength, conferring a higher resistance to cracks, thus enabling the production of nanocomposites with improved mechanical properties with these nanostructures [23].

**Figure 3:**
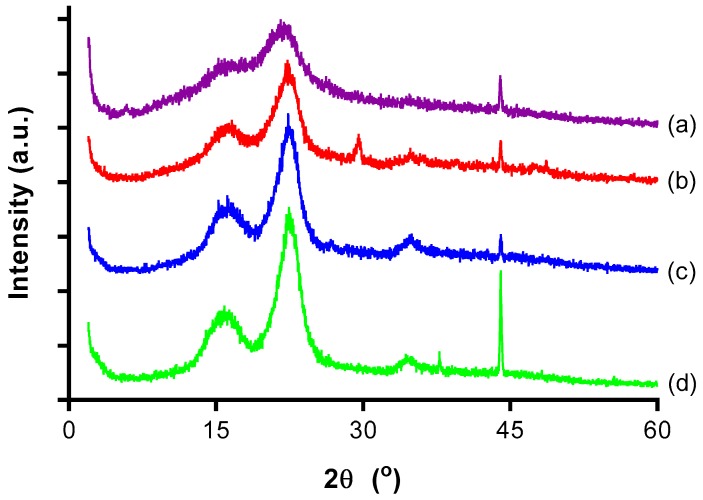
XRD curves of (a) OPMF, (b) alkaline treated OPMF, (c) bleached OPMF and, (**d**) CNC.

Figure 3 shows the X-ray diffractograms of (a) OPMF, (b) alkaline treated OPMF, (c) bleached OPMF and, (d) CNC. All the samples exhibited high peak intensity at a 2θ value of 22° related to their crystalline structure of cellulose. Alongside the presence of a broad peak at around 15°, which is related to the amorphous arrangement, OPMF can be indicated as having typical cellulose I structure [24].

With the main diffraction peak for cellulose I around 22° and the lowest intensity at a diffraction angle around 18°, the CrI was calculated and summarized in Table 1. The OPMF possesses the lowest CrI of 44.61% since it contains a high amount of amorphous regions. After chemical treatments via alkaline and bleaching, the crystallinity of the fibers had increased to 65.03% and 74.81% respectively. This was due to the removal of lignin and hemicelluloses that were attached to the cellulosic fibers.

The crystallinity index as calculated for the CNC showed an increase from the bleached OPMF to a value of 77.80% (Table 2). The increase of the crystallinity upon acid hydrolysis indicated the dissolution of an amorphous region of the cellulosic fiber. During the hydrolysis process, sulphuric acid attacks and penetrates the amorphous region of the cellulose, allowing the hydrolytic cleavage of glycosidic bonds and eventually releasing individual crystallites. In addition, growth and realignment of monocrystals may occur at the same time, contributing to the increase in cellulose crystallinity and narrowing of the diffraction peaks as shown in the XRD curve.

### 3.4. Thermogravimetric Analysis (TGA)

**Figure 4:**
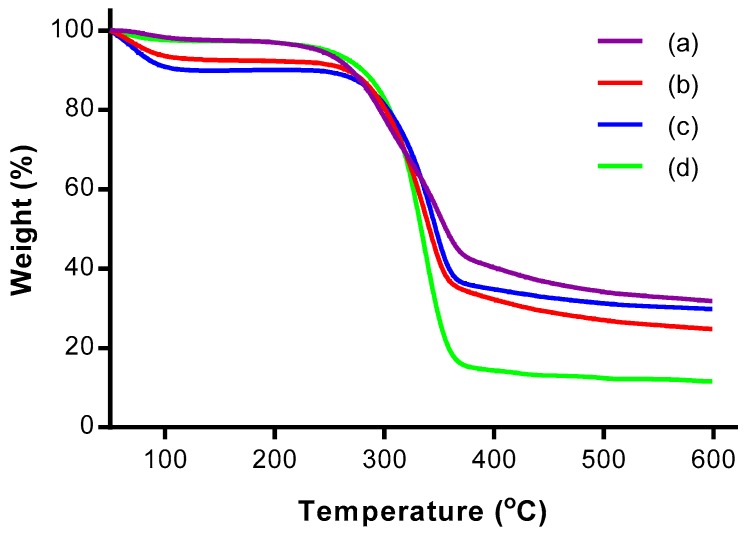
Thermogravimetric (TG) thermograms of (a) OPMF, (b) alkaline treated OPMF, (c) bleached OPMF and (d) CNC.

**Figure 5:**
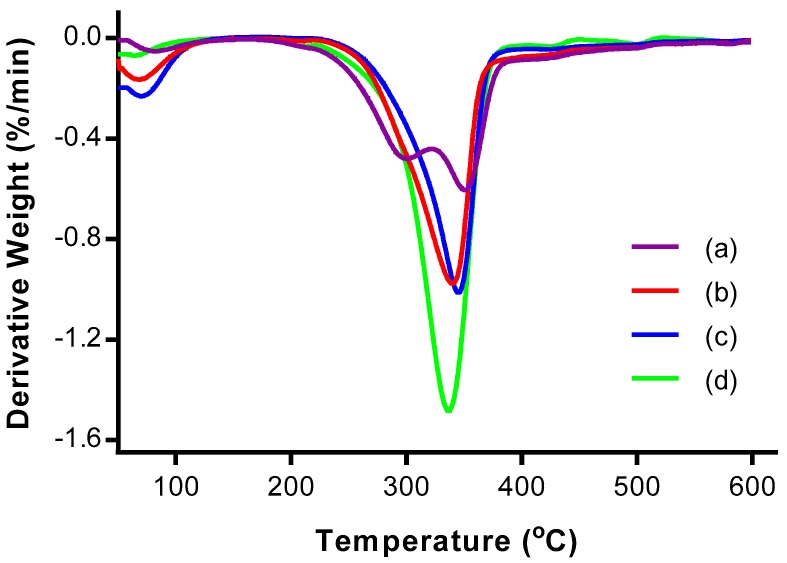
Derivative thermogravimetric (DTG) thermograms of (a) OPMF, (b) alkaline treated OPMF, (c) bleached OPMF and (d) CNC.

TGA was performed to study the thermal stability of the CNCs. Figure 4 and Figure 5 show the thermogravimetric (TG) and derivative thermogravimetric (DTG) thermograms, respectively, of OPMF, alkaline treated OPMF, bleached OPMF and CNC.

The TG and DTG thermograms of all the samples exhibited two main mass loss stages: at (i) high temperature range between 160 °C and 600 °C, and at (ii) low temperature range below 150 °C. In the low temperature range, a small mass loss (<10%) is detected for all samples due to the evaporation of physisorbed water from the surface of the samples [19]. In the high temperature range, the mass loss is relatively more (>59%) as a result of primary thermal degradation of the cellulosic samples. The thermal decomposition of samples involves the degradation of hemicellulose and lignin, and depolymerisation of cellulosic fibers.

The thermal decomposition of components in the samples can be allocated according to the temperature range of 160–250 °C and 300–350 °C, each representing the depolymerization of hemicellulose and the degradation of cellulose respectively [25]. After 380 °C, the residual decomposition products showed a slow degradation profile and reached a plateau. The residue that remained after heating the fiber up to 600 °C for all samples indicates the presence of carbonaceous materials in the OPMF.

From the DTG thermograms, only the thermogram representing OPMF has two peaks; one at 300.25 °C and the other at 350.34 °C. The two peaks correspond to hemicellulose and cellulose decomposition respectively. The disappearance of the broad peak in the range of 160–325 °C in the treated OPMFs has proven the removal of unstable hemicellulose ingredients during alkaline treatment. The removal of this peak can also indicate the partial degradation of cellulose since it degrades at high temperatures.

The degradation of the alkaline treated OPMF occurred from 217.20 to 530.35 °C, while that of bleached OPMF was in the narrower temperature range of 219.98–524.94 °C. Both of the temperature ranges can be attributed to the degradation of cellulose. The thermal stability of the cellulosic fibers is improved due to an increase in crystallinity and intermolecular hydrogen-bonded domains after the removal of amorphous components [26].

As for CNC obtained from OPMF, the thermal degradation occurred in the temperature range of 173.3–454.27 °C. This was due to the presence of sulphate groups (O–SO_3_) as a substitute for hydroxyl groups during acid hydrolysis, causing the sample to be less resistant to pyrolysis. As a result, dehydration of cellulose occurred, leading to release of water and catalyzed nanocrystal decomposition [27]. Furthermore, the lower thermal stability may be due to the smaller dimensions of CNCs, which provide a larger surface area accessible to heat treatment.

### 3.5. Scanning Electron Microscopy (SEM)

SEM was used to investigate the surface morphology of the OPMFs, from the original fibers to the fibers exposed to various treatments. The various treatments done on the fiber affect the fiber morphology, exposing its surface due to removal of some non-cellulosic, macromolecular components such as hemicelluloses, lignin, pectin, wax, and other impurities from the fiber causing fibrillation of fibers.

**Figure 6:**
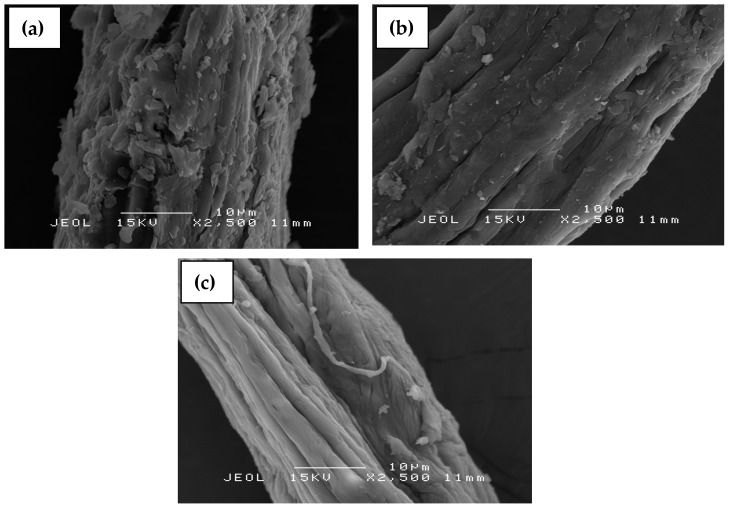
SEM micrographs of (**a**) OPMF, (**b**) alkaline treated OPMF and (**c**) bleached OPMF.

Figure 6 shows the SEM micrographs of (a) OPMF, (b) alkaline treated OPMF, and (c) bleached OPMF. These micrographs show the gradual changes of the effect of treatment on the surface of the fibers. It is shown that the OPMF surface is covered with impurities, wax, fatty substances and globular protrusions called “tyloses”. The significant changes of the fiber morphologies can be seen on the fiber after treatment with alkaline, in which it became cleaner and the roughness was reduced due to the removal of impurities from the fiber surface. Impurities such as wax and cuticle on the fiber surface were removed by the interaction with sodium the during alkaline treatment using NaOH [28].

In Figure 6c, it can be seen clearly that the fiber surface appears to be smoother than previous fibers, as more non-cellulosic components and impurities were removed during the bleaching process. Generally, the pretreatments of fiber through the alkaline treatment and bleaching process have broken the lignocellulosic complex, solubilized the lignin and hemicellulose to expose more porosity and surface area of the hidden cellulose. This has resulted in the cellulosic fibers aligning and distributing individually between each other, making them more accessible for the cellulose nanocrystal extraction process.

### 3.6. Transmission Electrion Microscopy (TEM)

**Figure 7:**
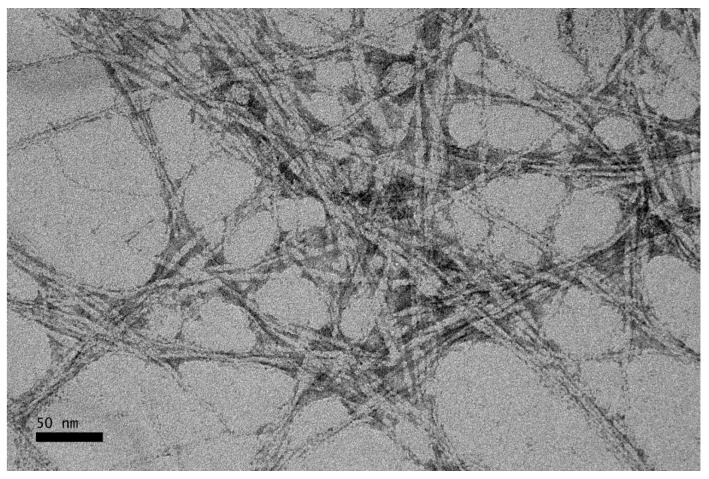
TEM micrograph of CNC extracted from OPMF.

TEM was used to study the morphology and nanometer scale of CNC. The acid hydrolysis process was expected to remove the amorphous part of the cellulosic fibers, while leaving the crystalline region intact and eventually reducing the size of the fiber to nanometer scale. Figure 7 shows the TEM micrographs of CNC with magnification of 50,000×. The CNCs had an average diameter of 4.52 nm. From the micrographs, it can be observed that the CNCs present are in rod-like shape with little agglomeration. The CNCs tend to agglomerate, probably due to surface ionic charge which had the crystallites stacked together as a result of the acid hydrolysis process [29]. The existence of the aggregates may also be a result of TEM sample preparation when the dispersing medium was removed [30].

## 4. Conclusions

CNCs have been successfully isolated from OPMF by acid hydrolysis, after the OPMF was exposed to an alkaline treatment and a bleaching process. The non-cellulosic materials of OPMF, such as lignin and hemicellulose were successfully removed after chemical treatments. CNCs showed increased crystallinity, indicating the exposure of the crystalline phase after the removal of lignin and hemicellulose via pretreatments of OPMF and the removal of the amorphous region of cellulose via acid hydrolysis. SEM images showed significant changes on the surface morphologies of the fibers. The fiber surfaces became smoother and eventually showed great reduction in diameter and size after acid hydrolysis. The isolated CNCs displayed their rod-like shape and further confirmed the size reduction of OPMF to a nanometer scale of 1*–*6 nm in diameter. The results obtained here suggest that OPMF is capable of being a source of fiber for the production of CNCs which can be used as reinforcing fillers in various industries. The successful utilization of OPMF shows how fiber resources can benefit the environment through their sustainability and biodegradability.

## Figures and Tables

**Table 1:** Chemical composition of oil palm mesocarp fiber (OPMF) and treated OPMF.

	OPMF	Treated OPMF
**Cellulose (%)**	32.22 ± 1.54	81.11 ± 0.45
**Hemicellulose (%)**	31.62 ± 0.46	12.65 ± 0.14
**Lignin (%)**	23.89 ± 1.12	5.09 ± 1.23

**Table 2:** Crystallinity index (%) of OPMF, alkaline treated OPMF, bleached OPMF and CNC.

Samples	2θ (Amorphous) (°)		2θ (002) (°)		CrI (%)
	Degree	Intensity (*I*_am_)	Degree	Intensity (*I*_002_)	
OPMF	18.76	298	21.58	538	44.61
Alkaline treated OPMF	18.44	214	22.28	612	65.03
Bleached OPMF	18.96	198	22.36	786	74.81
CNC	18.58	190	22.46	856	77.80
